# Epigenetic dysregulation of brainstem nuclei in the pathogenesis of Alzheimer’s disease: looking in the correct place at the right time?

**DOI:** 10.1007/s00018-016-2361-4

**Published:** 2016-09-14

**Authors:** A. Iatrou, G. Kenis, B. P. F. Rutten, K. Lunnon, D. L. A. van den Hove

**Affiliations:** 1 Department of Psychiatry and Neuropsychology, School for Mental Health and Neuroscience (MHeNS), Maastricht University, Universiteitssingel 50, 6200 MD Maastricht, The Netherlands; 2 University of Exeter Medical School, RILD, University of Exeter, Barrack Road, Devon, UK; 3 Laboratory of Translational Neuroscience, Department of Psychiatry, Psychosomatics and Psychotherapy, University of Wuerzburg, Fuechsleinstrasse 15, 97080 Würzburg, Germany

**Keywords:** Alzheimer’s disease, Locus coeruleus, Raphe nuclei, Epigenetics, DNA methylation, DNA hydroxymethylation

## Abstract

Even though the etiology of Alzheimer’s disease (AD) remains unknown, it is suggested that an interplay among genetic, epigenetic and environmental factors is involved. An increasing body of evidence pinpoints that dysregulation in the epigenetic machinery plays a role in AD. Recent developments in genomic technologies have allowed for high throughput interrogation of the epigenome, and epigenome-wide association studies have already identified unique epigenetic signatures for AD in the cortex. Considerable evidence suggests that early dysregulation in the brainstem, more specifically in the raphe nuclei and the locus coeruleus, accounts for the most incipient, non-cognitive symptomatology, indicating a potential causal relationship with the pathogenesis of AD. Here we review the advancements in epigenomic technologies and their application to the AD research field, particularly with relevance to the brainstem. In this respect, we propose the assessment of epigenetic signatures in the brainstem as the cornerstone of interrogating causality in AD. Understanding how epigenetic dysregulation in the brainstem contributes to AD susceptibility could be of pivotal importance for understanding the etiology of the disease and for the development of novel diagnostic and therapeutic strategies.

## Introduction

Alzheimer’s disease (AD) is a chronic, neurodegenerative disorder that currently accounts for 60–80 % of dementia cases [1, 2]. The prevalence of AD is expected to increase dramatically with the exponential increase in the aging population and a lack of effective therapeutic options. Recent evidence suggests that the incipient stages of the disease may start in young adulthood where they remain asymptomatic until advanced age [3, 4]. Throughout its progression, AD deprives patients of their quality of life, by negatively impacting upon emotional control, cognition, memory, and language skills, converting them to highly dependent reflections of their past selves, and substantially decreasing their life expectancy. The pathogenesis of AD is associated with amyloid beta (Aβ) plaques, which form degradation-resistant aggregates, and hyperphosphorylated tau protein that leads to the formation of intraneuronal neurofibrillary tangles (NFTs) [2, 5]. These two characteristic hallmarks are believed to lead to synaptic dysfunction and eventually neuronal cell loss, causing dramatic cortical and subcortical atrophy [6–9]. While the hypotheses about the preliminary appearance of one of the two hallmarks are raging, a definite mechanism has yet to be provided (i.e. [8, 10]). To date, the Aβ burden has mainly been associated with the neurobiological underpinning of AD, whereas tau pathology is positively correlated with the progression of cognitive deterioration in the patients [3, 10, 11].

In AD, individual disease risk is determined by genetic and environmental factors, as well as complex interactions between them. From a genetic perspective AD can be classified into two subtypes, familial or sporadic, and while the symptomatology and the progression of both forms are comparable, the etiology is fundamentally different [12]. Familial AD accounts for only 5–10 % of the disease cases and is related to the existence of genetic mutations in specific genes, such as those encoding amyloid precursor protein (*APP*) and presenilin (*PSEN*) *1* (*PSEN1*) and *PSEN2* [13–20], which are all involved in the production of Aβ. Sporadic AD is the most prevalent form of AD, usually occurs later in life (>65 years) and bares non-Mendelian traits. In recent years, common genetic variants have been robustly associated with sporadic AD via genome-wide association studies (GWAS) and subsequent meta-analyses ([21]; for specific GWAS results see [22–26]), although these only account for a third of disease susceptibility risk [21]. Therefore, more recent research efforts have focused on a potential role for epigenetic mechanisms in disease etiology [27].

To date, even though there is a strong association between hallmark appearance and the incidence of AD, the pathogenesis of the disease remains uncertain. Moreover, evidence has shown that some individuals may carry the most salient genetic risk factors for AD and also express profuse Aβ and tau pathology, but yet never develop the disorder [17, 28–30]. Strikingly, even monozygotic twins can have discordant AD outcomes [29], and as such it has been suggested that these phenomena could be explained by epigenetic mechanisms [27]. The epigenetic machinery induces reversible changes in gene expression via covalent interactions with mainly the chromatin components. These modifications in gene activity, while ever-changing, are more pronounced during development and remain more stable in differentiated cell types. Hence, normal dynamic changes in the epigenetic machinery are responsible for cellular development and differentiation, but also for transiently imprinting environmental, behavioral as well as social effects on gene expression, maintaining genomic homeostasis throughout the lifespan. The umbrella term, epigenetic modifications, covers a gamut of mechanisms, namely DNA modifications [5-methylcytosine (5-mC), 5-hydroxymethylcytosine (5-hmC), 5-formylcytosine (5-fC), and 5-carboxylcytosine (5-caC)], chromatin remodeling by means of remodeling complexes and post-translational histone modifications, and non-coding RNA [ncRNAs; long ncRNA (lncRNA), short ncRNA (sncRNA)]. Currently, the best-characterized epigenetic modifications are DNA modifications, with DNA methylation within CpG islands being the most extensively studied. Contrary to popular belief, DNA methylation is not solely associated with gene repression, but the differential effect on gene activity depends on the location of the epigenetic modification on the gene or its proximity [31]. Additionally, the newly characterized DNA modifications, 5-hmC, 5-fC, 5-caC, were originally thought to be transient marks in the demethylation pathway; however, recent evidence suggests that 5-hmC may represent an independent epigenetic mark and has been associated with active gene transcription [32]. In AD, recent epigenome-wide association studies (EWAS) have identified robust changes in DNA methylation patterns in specific genes; yet whether this remains a cause or a consequence of the disease is not currently known.

This review provides a thorough update on the fast-pacing advancements in (epi)genomic technology with a main focus on its application to AD-related research. Moreover, by reviewing recent evidence on the early involvement of the brainstem in the non-cognitive early symptomatology of the disease, it discusses the need to systematically assess epigenetic dysregulation in this brain region to identify novel dysfunctional pathways. Ultimately, this review aims to raise critical questions of temporal and spatial causality of AD pathogenesis and how the answer may be found in innovative brain structure targets with the assistance of state-of-the-art genomic technology.

## Epigenomic technology advancements in AD

Over the past decade, the number of publications investigating the role of epigenetic mechanisms in AD has dramatically increased, which have substantially contributed to our understanding of the disease (reviewed by Lardenoije et al. [17]). Major advances in genomic technology have helped overcome numerous hurdles that were faced in the early years of neuroepigenetic studies [27]. Such caveats involved the limited available techniques, the specificity and reliability of the epigenetic methodology used, as well as issues concerning genomic coverage, tissue cell-type composition, and sample sizes.

### Towards genome-wide sequencing of the AD brain

It is evident that genomic studies in AD have now progressed from restricted, targeted antibody-based techniques to genome-wide arrays and sequencing technology with single CpG site resolution. In 1995, the first empirical studies in AD used methylation-specific restriction enzymes and Southern Blot technique demonstrated hypomethylation in the *APP* gene promoter region [33]. Since then, several approaches have emerged, involving immunohistochemistry, methylation-specific polymerase chain reaction (PCR), bisulfite (BS) conversion, high performance liquid chromatography (HPLC), pyrosequencing and various methylation assays [34]. The imperative need for more concise and collective results shifted epigenetic research in AD to more systematic genome-wide approaches. In 2012, Baluski and colleagues were the first to utilize Illumina microarray technology, Infinium HumanMethylation27 Beadchip assay, enabling quantification of DNA methylation at >27,000 CpG sites, and detected AD-associated DNA methylation differences in the prefrontal cortex of late-onset sporadic AD patients in comparison to cognitively normal controls [35]. More recently, studies have employed the more extensive, current workhorse for epigenetic studies, the Infinium HumanMethylation450 BeadChip assay (450K), detecting >485,000 methylation sites at a single nucleotide resolution, covering approximately 1.5 % of total genomic CpG sites, mainly amidst promoter regions [26, 36–39]. The first two large-scale EWAS in AD identified overlapping differentially methylated CpG loci, namely *ANK1, RPL13, CDH23, and RHBDF2* [40]. This year, a further Illumina Infinium microarray (Illumina MethylationEPIC Beadarray) was launched, covering >850,000 CpG sites [41], although it has yet to be utilized in AD. The continuous advancements in microarray technology, combined with their cost effectiveness have made this approach the most widely utilized EWAS method in large sample cohort studies. However, such methodologies only cover a small percentage of CpG sites and thus whole-genome sequencing techniques remain the best option for in-depth genome-wide examination. Only recently, the first whole-genome bisulfite sequencing (WGBS) was conducted with much wider coverage than just the promoter proximal CpGs (20 % of total genomic CpG sites) [42]. In addition, the first low(er)-cost deep sequencing reduced representation bisulfite sequencing (RRBS) kit recently became available with a high coverage of up to 4,000,000 CpGs in human samples [43], and as such the use if these technologies in AD tissue is anticipated.

### Beyond DNA and CpG specific methylation

Epigenomic studies have been largely focused on DNA (cytosine) methylation, overlooking additional epigenetic signatures. To date, further methodological improvements have allowed the detection of demethylation marks (5-hmC, 5-fC, 5-caC), post-translational histone modifications as well as deregulated ncRNAs. New advancements have allowed the discrimination of 5-hmC and 5-fC by employing oxidative bisulfite sequencing (oxBS-Seq) and reduced bisulfite sequencing (redBS-Seq), respectively [44–47]. More recently, researchers have made use of this chemistry and coupled it with the 450K array, presenting the oxBS-450K method [48]. This method was used successfully to identify differential DNA hydroxymethylation patters across different anatomical region of the human brain but also and most importantly to accurately quantify “true” methylation levels that up until now were confounded by hydroxymethylation levels [49]. Application of oxBS-arrays or oxBS sequencing on human AD samples will hopefully not only highlight the importance of DNA demethylation in cognitive processes but also confirm the hypothesized crucial role of 5-hmC in AD as hinted by immunohistochemical studies [50–53] (reviewed in [54]). Whilst for the DNA modifications 5-fC and 5-caC, there are currently only two studies examining their levels in AD with disparate results; Condliffe et al. [51] did not detect AD-associated differences, whereas Bradley-Whitman et al. reported a decrease in the hippocampal area in preclinical AD samples [55]. Therefore, studies using redBS-Seq or comparable techniques will elucidate the levels of these modifications in AD at single nucleotide resolution.

While traditional epigenetic research has focused on methylation of a cytosine within a CpG dinucleotide, largely within CpG islands, more recent studies have begun examining intermediate/low CG-content regions as well as non-CpG DNA methylation. An increasing number of targeted AD studies nowadays examine the methylation status of more than just CpG-rich gene promoter areas [38]. The newly developed WGBS method provides adequate information about intergenic CpGs distal to gene promoters as well as non-CpG methylation [42]. Thus, it is expected that implementation of this technique in AD studies will contribute to a deeper understanding of DNA methylation to the pathophysiology and will highlight further regions on the genome that display differential DNA methylation in disease.

Although genome-wide histone modification analysis using chromatin immunoprecipitation (ChIP) techniques are available, to date no studies have used this approach in AD. In fact, only three published studies have directly connected histone modifications to AD. Zhang and colleagues found downregulated H3K18 and H3K23 acetylation when comparing temporal lobe samples from AD patients to those of controls using monitoring liquid chromatography-mass spectrometry [56]. Mastroeni and colleagues immunohistochemically detected aberrant extra-nuclear localization of H3K4 tri-methylation at the most incipient stages of the disease [57]. Whilst, Graff and colleagues detected increase in the protein levels of histone deacetylase 2 (HDAC2) in AD brains [58]. Finally, while micro-RNAs (miRNAs) are very well studied with targeted and genome-wide array-based methods, other ncRNAs have been generally understudied. Recently, though AD-associated lncRNAs have been identified for the first time by re-annotating previously probed uniquely mapped lncRNAs [59]. Among the most significantly dysregulated lncRNAs were n341006 and n336934, lncRNAs involved in protein ubiquitination and cholesterol homeostasis, respectively [59].

### Cross regional and blood differences in epigenetic modifications

One caveat when examining epigenetic as opposed to genetic variation is the need to investigate changes in a tissue-specific manner. To date, studies have largely utilized tissue from various cortical regions given that these are the site of neurodegeneration and dysfunction observed with advanced progression of the disease (hippocampus, frontal cortex dorsolateral prefrontal cortex, the entorhinal cortex, the superior temporal cortex, the medio-temporal gyrus, the superior temporal gyrus), although a handful have also included the cerebellum [36–38, 51]. The use of cerebellum is rather interesting in such studies as it is relatively spared from AD pathology, even at the late stages of the disease, and thus serves as an internal control tissue.

The investigation of tissue-specific epigenetic signatures in the brain allows the elucidation of the underlying mechanisms in the pathophysiology of AD, whilst interrogation of epigenetic variation in the blood is of pivotal importance to develop novel molecular biomarkers for the early diagnosis of AD. To date only a handful of studies have investigated DNA methylation changes in blood from AD patients; D’Addario’s team showed global DNA hypermethylation in blood samples of sporadic AD patients, while Lunnon et al. detected DNA methylation differences at specific loci in ante-mortem blood samples from sporadic AD patients [37, 66]. Altogether, these results encourage further research to identify AD-related epigenetic signatures as biomarkers in larger sample cohorts.

### Cell-specific epigenetic changes

While the identification of AD-related epigenetic changes in post-mortem brain tissue is highly important for a better understanding of the pathophysiology, the cellular heterogeneity constitutes a major caveat in interpreting the results. It is well described in the literature that AD-related neurodegeneration is highly specific towards selected neuronal cell types and is also accompanied by glial activation, which could confound the interpretation of epigenetic studies on brain tissue in AD. Steps to specify cell type composition can be taken in early experimental stages with a range of methods available to isolate specific cell types. Such methods involve density gradients, laser capture microdissection (LCM), fluorescent-activated cell sorting (FACS), magnetic affinity cell sorting (MACS) and, more recently, isolation of nuclei tagged in specific cell types (INTACT) [60, 61]. The INTACT method is specifically adapted for interrogating epigenetic marks ranging from DNA methylation to histone modifications by means of selectively capturing nuclei that express an antibody-tagged protein [60]. Hence, its application will be very fruitful for unravelling neuron or glial specific AD-related epigenetic signatures. LCM has recently been employed for the characterization of amyloid plaques [62] as well as gene expression via RNA sequencing comparing AD and control brain tissue [63]. Encouraging data from the latter study imply that LCM could be used for specific cell type isolation in epigenetic studies, since it does not appear to induce disease-unrelated transcriptional changes. Interesting applications of this methodology would be not only targeting the epigenetic profiling of neurons either in the vicinity of AD hallmarks (namely gliosis and amyloid plaques), or severely affected by tau pathology, but also assessing the differential epigenetic signatures of AD pathology spared neurons. Finally, in already collected datasets on unsorted tissue, bioinformatic analyses can also correct for neuronal/glia composition utilizing published algorithms [64]. This approach has already been used in the analysis of 450K array data generated in AD tissue enabling the researchers to control for cellular heterogeneity bias [37, 38].

### Sample size caveat; loophole through validation cohorts?

Regardless of the technological improvements that have assisted a deeper investigation of the epigenetic machinery in AD, there is still one caveat that persists; the sample size of the cohorts used. To date, there is a circumscribed amount of EWAS studies on AD [35–38, 65, 66], and only two of them have a sample size exceeding 100 [36, 37]. A fortunate phenomenon in the limited number of EWAS studies in AD, performed to date, is that a considerable number of epigenetic alterations have a replicable effect in independent cohorts from other studies (i.e. finding from [37] have been replicated by [36, 38]). The falling cost of whole-genome studies in combination with the exponential increase in high quality brain tissue available from brain banks worldwide will probably reinforce studies with larger sample size. Nevertheless, one should be cautious with the predilection of tissue from AD patients. It was recently suggested that DNA methylation profiles of various neurodegenerative disorders, including AD, involve similar early epigenetic-associated pathogenic mechanisms, which, over time evolve into divergent clinical cases with distinct molecular and cellular underpinnings [65]. This concept was also supported by the latent early life associated regulation (LEARn) model of Lahiri and colleagues which proposes that neurobiological disorders share a similar mechanistic etiology [67]. More specifically, according to this hypothesis, early life stressors modify the expression levels of disorder-associated genes, a change that is transiently maintained by epigenetic mechanisms and is shared among a wider spectrum of neurobiological disorders. The differential expression of these genes remains within physiological range, until, later in life, multiple “hits”, i.e. environmental agents, dietary factors, and lifestyle habits, accumulate, leading to aberrant-pathological changes in expression [67, 68]. Evidently, these notions should be taken into consideration for longitudinal studies in populations at high risk of developing AD [69]. Furthermore, research on incipient stages of AD might not be in conflict with other confounding factors, but as the pathology worsens, the epigenome seems to change dramatically. Moreover, recent evidence showed that once proper bioinformatic analysis is employed, i.e. correcting not only for technical issues and sex, but also for common neuropathologies seen in the elderly population—the total number of age-dependent CpG methylation profiles is reduced by approximately 40 % [70]. Therefore, large sample sizes with a thorough medical history of medication, information on concurrent neuropathologies as well as epigenetic-modifying environmental exposures, together with the reciprocal advances in bioinformatic analysis tools, would hone current EWAS studies in AD.

### Integrative genetic and epigenetic analyses

Even from the restricted number of EWAS studies thus far published, a common locus, BIN1 [26, 36], is found to overlap with GWAS results, leading the way for integrated analysis of genomic and epigenomic data that could essentially address causality in AD (for a thorough review see [71]). It has already been shown that genetic variants can influence DNA methylation [72]. In this respect, application of the Mendelian randomization (MR) method could strengthen the causal assumption, and help in elucidating the interplay among genetic variation, epigenetic modifications and environmental factors. For example, with the recently described two-step epigenetic MR method, first the causal impact of a risk factor in an epigenetic modification is interrogated using a genetic variant as intermediate for the risk factor, and then the causal effect of the investigated epigenetic change, is examined on the desired outcome (i.e. AD) [73]. To date, EWAS data can assist identifying the risk factor-epigenetic modification association at the first step and GWAS data can provide the genetic variant proxy. Interestingly, with the identification and study of methylation quantitative trait loci (mQTL) in the human brain [74], it will be possible to trace SNPs associated with methylation at specific genomic regions and use them as proxy [73]. An additional integrative analysis of genomic, epigenomic and enviromic data called longitudinal epigenome/envirome/exposome-wide association study (LEWAS) was suggested by Lahiri and Maloney [75]. The rationale of this approach is the combination of genomic information with repeatedly collected information of the patient’s envirome and the epigenome [75, 76]. Therefore, changes in epigenetic markers could be linked to the transient changes measured prior to the clinical manifestation of a disease [75]. All these approaches would allow the exploration of new disease mechanisms to ultimately start to answer the question: “Is epigenetic dysregulation a cause or consequence of AD?” Nevertheless, at this point, it is important to note that while the MR approach is feasible practically, LEWAS remains a rather theoretical method due to the in-depth interrogation of the patient’s environmental exposures as well as the high costs it would require to conduct such a study (reviewed by Maloney and Lahiri [76]).

One key issue that is yet to be addressed is the temporal and spatial causality of AD pathogenesis, for example whether the “state-of-the-art” technology that is being applied in the field is being done so in relevant brain regions at appropriate time points. Nowadays, ante-mortem AD diagnosis is mainly based on cognitive deficits associated with hippocampal and cortical dysfunction, as well as with imaging studies, primarily focused on the size of the hippocampus, which also makes these brain regions primary targets for GWAS and EWAS studies. Despite the catalytic involvement of hippocampal neurodegeneration and dysfunction in the progression of the disease, it is speculated that once the pathology has reached these structures, the deleterious effects on brain integrity are already irreversible [77]. Therefore, the interrogation of (epi)genetic modifications at that stage mainly contributes to a mechanistic understanding of the progression rather than the cause of the disease. A full mapping of epigenetic changes in a range of different brain structures at the appropriate stage(s) of disease is more likely to offer insight into the disease course, from the initial stages to the extensive neurodegeneration of cortical and subcortical areas. Such an approach could prompt early stage biomarkers and novel therapeutic targets for the most incipient stages of the disease, in addition to providing predictive models for the expansion of the disease.

## Brainstem: where it all starts?

Human AD pathology is primarily confined to the central nervous system (CNS) [78, 79]. There, the pathology propagates in a rather predictable, selective spatial and temporal manner with some regions being highly vulnerable to the aforementioned hallmarks at specific stages and others relatively resistant [7]. It is remarkable that the pathology vastly targets very specific neuronal types, which share long, late-myelinating and weakly myelinating axons [7, 8]. Thus, the earliest detection of abnormal hyperphosphorylated tau protein has been observed in the brainstem, and more specifically in the magnocellular nuclei of the basal forebrain, the raphe nuclei and the locus coeruleus (LC) [3, 4, 7, 8, 80–104]. From there, pathology propagates to highly vulnerable subcortical areas, i.e. the entorhinal cortex and hippocampus, and, subsequently, to high-order association areas of the neocortex [7, 85, 105]. Once cortical areas are affected, the curtailment of intellectual functions begins, gradually leading to deterioration or even loss of executive functions, annotating the clinical phase of AD.

The well-orchestrated propagation of hallmarks from subcortical to cortical regions has allowed staging of the various preclinical and clinical phases of AD and has facilitated the definition of neuropathological diagnostic criteria. Among the most widely used are the modified criteria based on NFT propagation described by Braak and Braak [106]. The original staging scheme of 1997 included four stages: Braak stage 0 (no NFTs); Braak stages I/II, with NFTs amidst the (trans)entorhinal cortex area; Braak stages III/IV, with NFTs expanding over to the hippocampus and the amygdala as well as cortical areas; and Braak stages V/VI, with pronounced NFTs over the isocortex [107]. In 2011, the aforementioned scheme was updated with the addition of the preclinical stages a–c and 1a–1b. Indicative of stages a-c is non-fibrillar abnormal tau pathology in the brainstem, mainly becoming traceable during teenage years [3]. Stages 1a–1b concern cases with early abnormal tau pathology at pyramidal cells in the transentorhinal cortex. Finally, extensive research complements the Braak staging providing associations with Aβ pathology measurements as well as AD clinical assessment tools, i.e. the mini-mental stage examination (MMSE) [7].

Increasing evidence that the brainstem may be the starting point of the propagation of AD pathology has triggered an ever-increasing scientific interest in the involvement of the brainstem in AD and numerous studies have investigated a central role of the brain serotonergic and noradrenergic systems in its pathophysiology (reviewed by [7, 87, 103]). The brainstem in AD patients was recently shown to be subjected not only to significant volume reductions, but also structural deformations in a magnetic resonance imaging (MRI) study [108]. Further, the early occurrence of various non-cognitive, behavioral and neuropsychological symptoms in AD, such as depression, general disturbances in mood, emotion, appetite, respiratory and circadian rhythm, suggests brainstem involvement, and more specifically that of the raphe nuclei and the LC [3, 87, 109]. Moreover, brainstem nuclei are affected by AD pathology, particularly tangles, in very early, presymptomatic stages [3, 7, 87, 103, 110, 111]. Interestingly, despite their vulnerability to tau pathology, the death of NFT-bearing neurons is not imminent during the presymptomatic stage and even at the final stages these neurons seem to be more resilient to degeneration [7, 92, 99, 103, 112–115]. Nevertheless, at that time, their function is highly impaired, impacting on the brain’s neurochemical balance [103].

### The raphe nuclei and AD

The raphe nuclei, and in particular the dorsal raphe nucleus (DRN) contains long projecting neurons that are abundant in serotonin (5-hydroxytryptamine; 5-HT), a monoamine neurotransmitter synthesized out of tryptophan [116]. The serotonergic system has been implicated in almost every type of basic physiological behavior, including appetite, sleep, emotional, cognitive as well as motor and neuroendocrine functions [116]. This widely distributed network in the brain mainly innervates the prosencephalon, including key areas for cognitive function, such as the frontal cortex, hippocampus, striatum, hypothalamus and amygdala [117, 118].

One study has found that more than 20 % of Braak stage 0 individuals and 100 % of Braak stage 1 individuals have detectable NFTs in the DRN, indicating that the DRN is affected by AD pathology even before the transentorhinal cortex [119]. Accordingly, it has been suggested that the development of pathology in the brainstem might trigger a transneuronal spread of NFTs changes to interconnected cortical brain areas affected at later Braak stages [110]. Even if tau “seeding” is still poorly understood, a suggested mechanism is that, once released, intracellularly formed tau aggregates extracellularly and is transferred to neighboring cells, thereby inducing the production of abnormal tau at those sites [120]. Several hypotheses on the formation and propagation of neurotoxic Aβ species have ensued from this hypothesized “seeding” effect. In particular, Braak and Del Tredici suggested that Aβ may originate from projection neurons with abnormal tau within the brainstem nuclei. Observations of accumulated toxic Aβ species in the vicinity of somatodendritic compartments of neurons as well as in the terminals of their axons in brain structures well-innervated by NFT-bearing 5-HT/NA projections could justify the fine pattern of Aβ propagation and suggests that toxic Aβ species are produced and released from such projection neurons [8]. Nevertheless, this hypothesis remains to be tested.

The severity of AD pathology in the DRN has been correlated not only with serotonergic denervation but interestingly also with behavioral changes in AD patients [103, 104, 121]. For example, the NFT-associated lesions that are present in the DRN even in the early phase, are largely held responsible for explaining mood symptoms such as depression and aggression, in prodromal AD [104]. Plaque and NFT load in the DRN and the median raphe nuclei (MRN) of AD patients has been shown to correlate with the progression of clinical symptoms [121, 122]. Additionally, a dysregulated serotonergic system has been linked not only to cognitive decline, but also to disturbances in the circadian rhythm seen in prodromal AD stages [123, 124].

From an anatomical point of view, post-mortem immunohistochemically stained AD brain samples (Braak stage V and VI) have shown a decreased number of serotonergic neurons in the DRN and the MRN [103]. This observation was recently replicated and enhanced with correlation analysis that exhibited an age-dependent 5-HT cell loss in particular nuclei [7, 103]. Interestingly, there seems to be a predilection for neurodegeneration in the caudal part of the DRN, which predominantly projects to the septum and the hippocampal area [98, 121]. Supporting evidence for the involvement of the 5-HT system in AD has been provided by imaging studies. Positron emission tomography (PET) studies have found that 5-HT_1A_ receptors were reduced in the hippocampi and raphe nuclei and that the decrease was strongly correlated with deterioration in the MMSE scores [125]. Moreover, while in MCI patients a hippocampus-specific increase in 5-HT metabolism and receptors (5-HT1A) has been observed, in advanced stages of AD, serotonergic receptors are dramatically downregulated in cortical areas [103]. Interestingly, functional genetic coding variants in the brain-specific tryptophan hydroxylase-2 (TPH2) and the 5-HT transporter (5-HTT) have been significantly associated with frontal lobe symptoms in AD [126].

Complementary research on the role of the serotonergic system in AD has yielded interesting results concerning 5-HT system function and AD pathology. Preclinical studies have demonstrated that an increase of 5-HT levels via, e.g. pharmacological activation attenuates Aβ pathology. Both acute and chronic administration of selective serotonin reuptake inhibitors (SSRIs) induces reduction in the production of toxic Aβ species in brains of APPswe/PS1dE9 mice, a widely used an AD mouse model [127, 128]. The acute administration of SSRIs is directly impacting on Aβ synthesis rather than the clearance rate of the plaques, the main mechanism of action of the ineffective drug bapineuzumab, a humanized monoclonal antibody targeting Aβ, developed for the treatment of AD [129]. In addition, intrahippocampal infusion of 5-HT as well as dietary enrichment of tryptophan in the same AD mouse model is associated with a reduction in the formation of Aβ plaques. Furthermore, treatment of non-cognitive impaired elderly participants with SSRIs for five consecutive years has been associated with less cortical amyloid deposition as revealed by a positron emission tomography (PET) study. Moreover, a reduction in the production as well as levels of Aβ was detected in the cerebrospinal fluid (CSF) of healthy volunteers treated with citalopram, a commonly used SSRI [128]. Finally, while 5-HT1A, 5-HT4 and 5-HT6 receptor ligands are known to modify cognitive functions [130], they were also shown to favor the production of non-amyloidogenic Aβ precursors that do not aggregate, with administration of 5-HT4 receptor agonists increasing the levels of soluble APP (sAPP-α) [131].

### The locus coeruleus and AD

The LC is the principal site for brain synthesis of noradrenaline (NA). NA is a catecholamine synthesized from tyrosine by a series of enzymatic steps that lead to the formation of dopamine, which is finally converted to the final product by dopamine beta-hydroxylase (DBH) [103]. Similar to the 5-HT system, the NA system consists of long projection neurons that are widely distributed throughout the brain [8]. Nevertheless, patterns of regional specificity arise as the frontal and parietal cortices are richly innervated [132]. Functionally, NA has been implicated in wakefulness and attention as well as the endocrine response to stress, while more recent evidence has also linked it to cognition, pain, aggression as well as energy homeostasis and blood flow control [132].

With regard to the role of the LC in AD, Braak and colleagues revealed that tau pathology is present in the LC prior to any other structure and even before any clinical symptoms or amyloid pathology manifestation was evident [3]. Histopathological observations of the LC using post-mortem AD brains have identified signs of atrophy, including swollen cell bodies, contracted dendrites and significantly decreased detection of NAergic markers [90, 91, 103, 105, 112, 133–135]. The deformation of the LC and the associated impairment in NA neurotransmission have been linked to the onset, severity, disease duration, speed of cognitive decline as well as with the appearance of AD pathology [103]. Concerning the latter, loss of NA neurons has been associated with increased Aβ deposition as well as an increased amount of cortical NFTs, strongly supporting the seeding-like propagation pattern previously suggested [8].

The noradrenergic system appears to be highly dysregulated in AD. Post-mortem studies have demonstrated reduced NA synthesis and availability in the frontal and temporal cortex as well as in the hippocampal area [90, 136, 137]. The rate of reduction in NA levels has been positively correlated with the severity of AD [133, 136, 138, 139]. Additionally, Vermeiren and colleagues showed significantly decreased levels of 3-methoxy-4-hydroxyphenylglycol (MHPG), a metabolite of NA degradation, in the prefrontal cortex of AD patients suffering from depression [140]. Nevertheless, other studies have reported an increase in NA and MHPG plasma and CSF levels solely in advanced AD cases [141, 142]. Another study did replicate the increased NA concentration in the CSF but failed to detect changes in the levels of MHPG [143]. Notably, one should be cautious interpreting results from CSF studies to brain function, as NA normally cannot cross the blood brain barrier (BBB), while its metabolites like MHPG, are able to do so [144]. Thus, CSF concentrations of MHPG reflect a sum of central and peripheral levels and are unlikely to reflect the most conclusive markers of the disease progression [145].

Studies have also indicated a decrease in DBH activity in the neocortex and the hippocampus of post-mortem tissue from patients at early AD stages [146]. This observation has been recently replicated by Mustapic and colleagues who additionally showed a gradual decrease in enzymatic activity with the progression of the disease and deterioration of cognitive functions [147]. Furthermore, the reported decrease could explain the decreased NA levels and the loss of noradrenergic neurons [147].

Restoration of NA levels via, e.g. exercise or pharmacological manipulation has shown beneficial effects on cognition in AD. Segal and colleagues demonstrated that exercise-mediated activation of the NAergic system can enhance memory consolidation in MCI patients and controls [148]. Moreover, administration of l-threo-dihydroxyphenylserine (l-DOPS), a prodrug for NA, enhances contextual and recognition memory in NA-deficient mouse models. Moreover, once administered to AD mouse models, restoration of spatial memory performance as well as a reduction in amyloid plaque number and size in the cortex and hippocampus were observed [149, 150].

## Looking to the future

Despite the increasing interest in brainstem dysfunction in AD, it still remains poorly understood whether the previously described structural, chemical, and functional alterations are causally involved in the pathogenesis of AD or whether they merely represent a consequence of the deleterious progression of the disease, or an epiphenomenon. The appearance of tau pathology with the early non-cognitive symptomatology suggests that the brainstem may reflect the initial structure affected by AD pathology in the brain. Bearing in mind the functional importance of the raphe nuclei and the LC as signaling hubs of top-down neuromodulatory input to high-order cortical areas as well as their vulnerability to AD neuropathology, it is tempting to speculate that they have a crucial role in the etiopathogenesis of AD. Furthermore, the lack of genetic attributes interlacing dysregulations in brainstem-specific neurotransmission with AD progression or pathology may furthermore, hint at an environmental and/or epigenetic involvement. Indeed, both the nuclei have been repeatedly investigated as targets of epigenetic control in various developmental stages as well as in neurological disorders. The noradrenergic developmental genes of the LC for instance have been reported to be under epigenetic control suggesting the vulnerability of the nucleus to environmental input [151, 152]. Moreover, it has been previously shown that functioning of the, i.e. 5-HT system is sensitive to gene-environment interactions (e.g. stress) [153–156]. Thus, the sensitivity of both the nuclei to environmental stimuli and epigenetic regulation in combination with the evidence that the brainstem is one of the first structures to present AD pathology and that robust epigenetic changes are seen in the latter effected cortical regions in AD offer fertile ground for further research into studying epigenetic dysfunction in the brainstem in the most incipient stages of AD.

The complex and yet elusive nature of sporadic AD allows for various hypotheses to explain the pathogenesis of the disease. To date, none of these hypotheses have been confirmed despite the advancements in genomic technology that provide deeper insight into the molecular underpinnings of AD. A possible reason is the temporal discrepancies between the biological and the clinical onset of the disease. Furthermore, the majority of research studies focus on brain structures vastly associated with the clinical phase of the disease thereby overlooking the preclinical manifestations of pathology. Such studies are invaluable in enriching the fundamental knowledge about the pathophysiology during the progression of AD, but it is unlikely that they will result in any of the two imperative societal needs: early, reliable, non-invasive and inexpensive biomarkers and effective treatment options that target the disease in its most incipient stages, much earlier than the first manifestations of cognitive curtailment.

Peripheral disease-associated epigenetic signatures have already been successfully employed as diagnostic tools for different cancers, and are currently being studied in neurological/psychiatric disorders [157–160]. As mentioned above, the advancements in genomic technology allows for high throughput interrogation of the epigenome and the extensive study of the correlation between brain and blood epigenetic signatures will contribute to the emergence of non-invasive and inexpensive biomarkers. Thus, epigenetic profiling of the brainstem of (sporadic) AD patients and its pairing with blood epigenetic signatures in the same individuals could potentially lead to the discovery of novel biomarkers that are able to detect either subtle changes at the very early stages of the disease, when the pathology is believed to be still reversible, or even an early peripheral response to AD pathology. Yet another exceedingly valuable asset of the study of epigenetic dysregulation in the brainstem is the fact that various pharmacological interventions impacting on either the epigenetic machinery ([161]; reviewed by Maloney and Lahiri [76]) or the 5-HT/NA system have already been developed and clinically approved; hence they could be implemented rapidly as a novel intervention for AD. Collectively, scrutinizing the interactions between the early AD-affected brainstem and the local epigenetic modifications will be of pivotal importance not only for understanding the pathogenesis of AD and the causal or consequential relationship of epigenetic alterations with AD, but also for the development of highly demanded early, reliable biomarkers and novel therapeutic strategies.

## Conclusion

The preclinical manifestations of AD, governed by non-clinical symptoms, suggest a crucial involvement of brainstem nuclei in the pathophysiology and most importantly in the pathogenesis of AD. Meanwhile, distinct, yet consistent, epigenetic signatures emerging from EWAS studies indicate a central role for the epigenetic machinery in the progression of AD. To date, while the exponential increase of AD-related research lines offers disparate interpretations in the cause of AD, the lack of effective diagnostic and/or therapeutic tools suggest that the etiology of the disease remains shrouded. With this review, we wish to perturb the status quo of AD, that is the genomic and epigenomic interrogation of brain regions like the hippocampus and cortex. We suggest that the temporal and spatial manifestations of the disease should be aligned and thus advocate that the two key nodes of the early stages of AD should be scrutinized. The pairing of brainstem pathology with deviant epigenetic regulation, indicative of the incipient stages of AD pathology, could serve as excellent candidate targets for further research that could lead to the development on early biomarkers as well as early treatment alternatives that could halt or even reverse the deleterious progression of AD.
